# Safe and effective use of rivaroxaban for treatment of cancer-associated venous thromboembolic disease: a prospective cohort study

**DOI:** 10.1007/s11239-016-1429-1

**Published:** 2016-09-30

**Authors:** Simon Mantha, Eva Laube, Yimei Miao, Debra M. Sarasohn, Rekha Parameswaran, Samantha Stefanik, Gagandeep Brar, Patrick Samedy, Jonathan Wills, Stephen Harnicar, Gerald A. Soff

**Affiliations:** 1 Memorial Sloan Kettering Cancer Center, 1275 York Ave, New York, NY 10065 USA; 2 Washington Hospital Center, Washington, DC USA

**Keywords:** Anticoagulation, Cancer, Venous thromboembolism, Rivaroxaban

## Abstract

Low-molecular weight heparin (LMWH) has been the standard of care for treatment of venous thromboembolism (VTE) in patients with cancer. Rivaroxaban was approved in 2012 for the treatment of pulmonary embolism (PE) and deep vein thrombosis (DVT), but no prior studies have been reported specifically evaluating the efficacy and safety of rivaroxaban for cancer-associated thrombosis (CAT). Under a Quality Assessment Initiative (QAI), we established a Clinical Pathway to guide rivaroxaban use for CAT and now report a validation analysis of our first 200 patients. A 200 patient cohort with CAT (PE or symptomatic, proximal DVT), whose full course of anticoagulation was with rivaroxaban, were accrued. In competing risk analysis, primary endpoints at 6 months included new or recurrent PE or symptomatic proximal lower extremity DVT, major bleeding, clinically-relevant non-major bleeding leading to discontinuation of rivaroxaban, or death. In competing risk analysis, the 6 months cumulative incidence of new or recurrent VTE was 4.4 % (95 % CI = 1.4–7.4 %), major bleeding was 2.2 % (95 % CI = 0−4.2 %) and all-cause mortality 17.6 % (95 % CI = 11.7–23.0 %). In this cohort of 200 patients with active cancer and CAT the rates of new or recurrent VTE and major bleeding were comparable to the cancer subgroup analysis from the EINSTEIN studies. The results of our Clinical Pathway provide guidance on Rivaroxaban use for treatment of CAT, and suggest that safety and efficacy is preserved, compared with past-published experience with LMWH.

**Electronic supplementary material:**

The online version of this article (doi:10.1007/s11239-016-1429-1) contains supplementary material, which is available to authorized users.

## Introduction

Cancer and its treatment are commonly complicated by venous thromboembolic (VTE) episodes, which remain a leading cause of morbidity and mortality in the cancer patient [1–3]. Management of cancer-associated thrombosis (CAT) remains problematic. Two randomized trials showed superior efficacy of low-molecular weight heparin (LMWH) to vitamin K antagonist (VKA) for management of cancer-associated thrombosis (CAT), [4, 5] thus establishing LMWH therapy as the standard of care [6]. However, LMWH therapy requires uncomfortable injections on a daily or twice daily basis, as well as substantial cost, even for currently available generic forms [7].

Recently, several new direct oral anticoagulants (DOAC’s) have become available for the treatment of VTE, as well as for other anticoagulation indications [8–12]. Rivaroxaban is a direct inhibitor of activated coagulation factor X and received approval in 2012 for treatment of VTE, following the EINSTEIN-PE and EINSTEIN-DVT trials [8–12]. The EINSTEIN trials compared rivaroxaban with a VKA in a general medical population, demonstrating non-inferiority of efficacy and significant reduction in major bleeding [8–12]. However, the EINSTEIN trials do not provide sufficient guidance for use of rivaroxaban in CAT. The control arm in EINSTEIN was a VKA, no longer considered standard of care for CAT. Secondly, only about 5 % of the patients in EINSTEIN had active cancer, and the nature and severity of those cancer subgroups may not be truly representative of the broader cancer population. Lastly, the EINSTEIN trials did not address the specific situations that arise during the course of cancer therapy such as transient thrombocytopenia, possible absorption and excretion defects, and interruptions for invasive procedures.

It is premature to extrapolate the EINSTEIN results to the general cancer population. This leaves a “knowledge gap,” where rivaroxaban is approved for treatment of VTE, but there has not been sufficient guidance on safe and effective use in the CAT specific population. To address this knowledge gap, the Hematology/Anticoagulation Management Service at Memorial Sloan Kettering Cancer Center, in consultation with expert pharmacists and published literature, derived a Clinical Pathway for rivaroxaban at the end of 2013. The Clinical Pathway addresses issues specific to the cancer population, including potential absorption defects, excretory organ function, drug interactions, thrombocytopenia, and bleeding risk. We now report on an initial 200 patient cohort with CAT, managed with the Clinical Pathway, conducted under an institutional Quality Assessment Initiative (QAI).

## Materials and methods

### Clinical pathway

The Clinical Pathway (Appendix 1) was devised by the authors (SM, SH, and GAS) and served as a guide for treatment of venous thromboembolic events (VTE) with rivaroxaban within Memorial Sloan Kettering (MSK). The Pathway largely reflected the FDA approved medication guide, but also accounted for anticipated situations arising within the course of cancer care. The usual starting dose was 15 mg orally twice daily for 3 weeks followed by 20 mg daily. For cancer patients aged 75 years or older the dose was reduced to 10 mg twice daily for 3 weeks followed by 15 mg daily based on an anticipated decrease in drug clearance for this demographic group.

Several relative contra-indications were established as part of the Clinical Pathway. Creatinine clearance <30 mL/min, liver function tests greater than three times the upper limit of normal, expected malabsorption at the level of the stomach or small bowel, active genitourinary (GU) or gastrointestinal (GI) lesions, untreated primary central nervous system neoplasm, a body weight <50 or >150 kg, the use of any antiplatelet agent other than aspirin 81 mg daily and any significant drug interaction all constituted relative contraindications to the use of rivaroxaban. A platelet count of 50,000/mcL or less, but ≥25,000/mcL led to a dose reduction, while the drug was held for a platelet count below 25,000/mcL. Some patients were treated with rivaroxaban despite relative contraindications. All patients who received rivaroxaban for CAT were included in the cohort analysis, regardless of compliance with the Clinical Pathway.

### Patients and outcomes

This project was conducted as an MSK Quality Assessment Initiative. The pre-specified goal was to track an initial cohort of 200 patients with cancer-associated pulmonary embolism (PE) or symptomatic proximal deep vein thrombosis (DVT), with a plan for at least 6 months of rivaroxaban treatment. Within this initiative, all patients who have had a rivaroxaban order on or after January 1, 2014 are entered in the database. The electronic medical record (EMR) is examined on all patients for cancer type, stage, initial thrombosis or other anticoagulation indication, and rivaroxaban dose and timing. We identified the initial 200 patients who had sustained an acute cancer-associated PE or symptomatic, proximal DVT and had been started on rivaroxaban for this indication. A VTE was deemed cancer-associated if the patient had active cancer, was receiving cancer-directed medical or radiation therapy, or the thrombotic episode had occurred within 30 days of cancer-related surgery. We included patients who had received up to 3 days of a parenteral anticoagulant as initial treatment prior to change to rivaroxaban. Patients with localized squamous cell carcinoma or basal cell carcinoma of the skin were not included in this cohort analysis. All study patients were followed at MSK after their initial diagnosis of CAT, even though they have received some of their cancer treatment locally as well. Patients who left MSK before the end of the 6-month mandatory observation period were censored as of the time of their last visit with us, or last available medical record from outside provider.

All clinical notes were reviewed by a combination of automated text search and review by an investigator, to detect bleeding or thrombotic episodes. The list of keywords is provided in Appendix 2. All clinical notes containing any keyword were first identified by an automated text search then reviewed by a physician (SM, GAS or EL). Outside medical records were read manually. Endpoints of interest included recurrent VTE (definition derived from the one used in the CLOT trial [4]), major bleeding (as defined by the International Society on Thrombosis and Haemostasis [13]), clinically-relevant non-major bleeding (CRNMB) leading to discontinuation of rivaroxaban, and death from any cause.

The R statistical software platform was used along with the TM package to process the clinical notes. All-cause mortality was also assessed based on the EMR. The date of transfer to hospice for terminal care was entered as the date of death due to difficulties assessing outcomes after such a change in plan of care.

The endpoint assessment on the 200-patient cohort was conducted when the final patient reached an endpoint or had been observed for at least 6 months.

### Statistical analysis

We initially conducted univariate analysis in order to assess the characteristics of cohort members along with time-to-event information based on the cumulative incidence for competing risks. Recurrent VTE, major bleeding, CRNMB leading to discontinuation of rivaroxaban and death from any cause were considered competing risks for the purpose of the analysis. The R 3.2.3 for Windows software platform was used, along with the Survival package.

## Results

Recruitment started on January 1, 2014. The first and last individuals to enter the cohort did so on January 25, 2014 and May 19, 2015 respectively. Patient characteristics are shown in Table 1, including the distribution of cancer types. The core cohort consisted of 200 patients, of whom 136 had sustained a PE (with or without DVT) and 64 had experienced a symptomatic, proximal lower extremity DVT as the index event. 183 patients had a solid tumor and 17 patients had a hematological malignancy. Stage was advanced for most cohort members, with 23 cases of stage III and 142 cases of metastatic disease for solid tumors (excluding brain primary). The mean age was 63 years, with 40 % of patients being male.

**Table 1:** Characteristics of patients

Characteristic	N
Gender	
Male	80
Female	120
Event type	
PE, with or without DVT	136
Proximal, symptomatic lower extremity DVT	64
Cancer stage (of solid tumors, excluding brain)	
0	3
1	5
2	7
3	23
4	142
Cancer types	
Pancreas	34
Gynecological	26
Lung	23
Breast	22
Genitourinary/prostate	21
Colorectal	18
Hematological	17
Stomach/esophagus	6
Other	33

*PE* pulmonary embolism, *DVT* deep vein thrombosis

### Primary endpoints

As of November 17, 2015 all patients in the cohort either had sustained an endpoint, had been observed at least 6 months on anticoagulation, or were censored as of the time of their last visit at MSK if they transferred their cancer care to another institution. No patient was lost to followup. At the 6 months time-point, the EMR was queried for clinical notes and the algorithm described above was applied in order to detect pertinent thrombotic and bleeding episodes. In the first 6 months of treatment, there were eight cases of recurrent VTE, four cases of major bleeding, seven cases of CRNMB leading to discontinuation of rivaroxaban and 31 deaths.

**Fig. 1:**
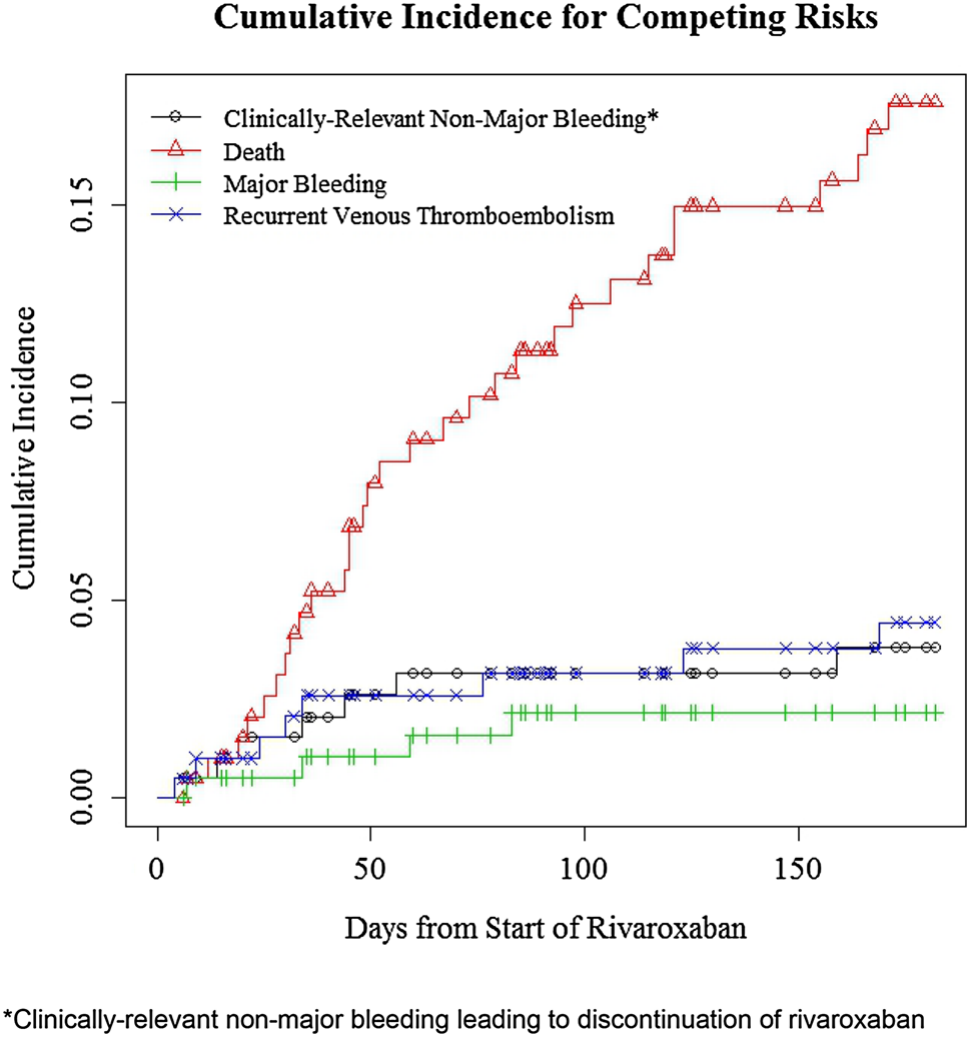
Cumulative incidence for competing risks

We reviewed the last notes and death notification for all 31 deceased individuals. In 24 cases, patients had been transferred to hospice, at which point rivaroxaban is usually discontinued and scant data exists about the cause of death. For the seven individuals who did not die under hospice care, a review of the record did not reveal any notion of sudden death or major bleeding. The records recorded deaths as “cancer-related” in 19 cases and “unknown” in all other instances. Other notable events, not included as primary endpoints, included four instances of tumor associated visceral vein thrombosis, one left hepatic vein thrombosis, and one ischemic stroke in patients on rivaroxaban. Eight patients had their dose of rivaroxaban decreased following the occurrence of an episode of CRNMB. Of these, there was one further major bleed and one CRNMB leading to discontinuation of rivaroxaban on the reduced dose. The 6-month cumulative incidence estimates for competing endpoints were; recurrent VTE, 4.4 % (95 % CI = 1.4–7.4 %); major bleeding, 2.2 % (95 % CI = 0−4.2 %); CRNMB leading to discontinuation of rivaroxaban, 3.8 % (95 % CI = 1.0−6.5 %); all-cause mortality, 17.6 % (95 % CI = 11.7–23.0 %). The plots for the cumulative incidence functions are shown in Fig. 1.

We performed a subgroup analysis of the 39 patients aged 75 years or older. The 6-month cumulative incidence values for recurrent VTE, major bleeding, and CRNMB leading to discontinuation were 5.3 % (95 % CI = 0−12.1 %), 2.6 % (95 % CI = 0−7.4 %) and 3.0 % (95 % CI = 0−8.7 %) respectively. These results were comparable to those of patients under the age of 75 years.

A total of 27 patients went off rivaroxaban prior to reaching a primary endpoint or 6 months of planned anticoagulation. The drug was stopped due to an upcoming surgical procedure in eight cases (and not restarted), for a medical reason in 18 cases, and following the patient’s wishes in one case. Slightly more than half of patients (105 individuals) were observed for the full 6 months, with an observation time range of 4–182 days for the whole cohort.

## Additional events

### Invasive procedures

There were 70 invasive procedures during the rivaroxaban treatment period (Table 2). In 59 procedures rivaroxaban was held for at least 48 h, and in six procedures it was held for 24 h. Rivaroxaban was not held in four cases: one was a diagnostic endoscopy, and three were emergency procedures. Documentation of rivaroxaban management was unclear in one additional case.

**Table 2:** Management of rivaroxaban anticoagulation in the setting of thrombocytopenia, renal insufficiency, liver dysfunction and invasive procedures

Episodes of rivaroxaban interruption/dose adjustments	Rivaroxaban dose-reduced	Rivaroxaban held	No change in dosage	Major bleeding	CRNMB^a^	Recurrent VTE	Death or hospice
Platelet count <50,000/mcL (N = 11)	1	7	3	0	0	0	0
Creatinine clearance <30 mL/min (N = 7)	0	2	5	0	0	0	1
Elevated liver enzymes (AST, ALT or bilirubin >3 × upper limit of normal) (N = 18)	0	6	12	0	1	0	1
Interventions^b^ (N = 70)	0	65	4	0	0	1	3

^a^CRNMB leading to discontinuation of Rivaroxaban

^b^The exact management approach is unknown in the case of one intervention

No major bleeding episode occurred during the interruption of rivaroxaban, within 7 days of the procedure. Only one patient developed a recurrent VTE following interruption for a procedure. That patient was a 52-year-old woman with stage IV ovarian cancer who had multiple discontinuations of rivaroxaban for debulking surgery and subsequent abscess drainages. Three days after an abscess drainage, prior to restarting rivaroxaban, she developed a symptomatic popliteal vein DVT. Of note, this patient was heterozygous for both factor V Leiden and prothrombin G20210A, with a history of multiple thromboses prior to her cancer, and therefore was at particularly high risk for VTE.

### Thrombocytopenia

There were 11 episodes of thrombocytopenia (platelets <50,000/mcL) in ten patients (Table 2). Rivaroxaban was held in seven episodes, and dose-reduced in one episode. In three episodes rivaroxaban dose was not adjusted, two of those cases only a single platelet count below 50,000/mcL and one patient had already been on reduced dose of rivaroxaban. There was no MB, CRNMB leading to drug discontinuation, death or recurrent VTE associated with an episode of thrombocytopenia.

### Renal insufficiency

Transient renal insufficiency, as defined by a creatinine clearance of <30 mL/min (using Cockroft–Gault equation [14]) was observed in seven episodes in five patients during rivaroxaban treatment (Table 2). Rivaroxaban dose was held in two episodes, and not altered in five episodes. None of the patients experienced a major bleed, CRNMB, or recurrent VTE event within 7 days of such an episode.

### Elevated liver enzymes

There were 18 episodes of elevated liver enzymes (aspartate transaminase, alanine transaminase or bilirubin >3 times the upper limit of normal) in 14 patients (Table 2). Rivaroxaban dose was held through six episodes and not modified in 12 episodes. One patient experienced CRNMB, which led to discontinuation of rivaroxaban. No patient experienced a MB or recurrent VTE event within 7 days of such an episode.

## Discussion

This is the first reported prospective cohort study of rivaroxaban therapy, specifically targeting CAT. In this cohort of 200 patients with active cancer and a new VTE episode, rivaroxaban use was found to be safe and effective when using a Clinical Pathway which accounts for situations specific to the oncology setting. Acknowledging differences in methodology, the risk of recurrent VTE and major bleeding in our cohort with rivaroxaban compared well with results from prior randomized trials using a LMWH for treatment of cancer-associated VTE. In two published trials of dalteparin, at 6 months the risk of recurrent VTE was 8.7 and 9 % and the risk of major bleeding was 6 and 10.2 % [4, 15]. In regards to this prior evidence base, 78 % of patients with a solid tumor in our cohort had metastatic disease versus 67 % for the CLOT study [4]. This suggests that patients in our group had a similar if not worst cancer comorbidity status compared to that of the CLOT trial.

Two recent papers have reported cancer subgroup analysis of rivaroxaban and apixaban for treatment of VTE. Prins and colleagues reported cancer subgroup analysis of the EINSTEIN studies of rivaroxaban for treatment of VTE, indicating similar rates of recurrent VTE (5 %) and major bleeding (2 %) to our new findings [16]. And more recently, Agnelli and colleagues performed a cancer subgroup analysis of treatment of VTE with apixaban in the AMPLIFY study, also showing comparable rates of recurrent VTE and major bleeding [17]. But both of those studies encompassed general medical populations with VTE, and the cancer subgroups were not the primary focus, and likely did not represent the full spectrum of cancer stages.

Our Clinical Pathway and cohort study of rivaroxaban for CAT only allow for indirect comparison with the past CLOT and DALTECAN studies of LMWH. But there does not appear to be evidence of loss of safety or efficacy by use of rivaroxaban for treatment of cancer associated VTE. And our all-cause mortality at 6 months of 17.6 % was lower than in the LMWH studies [4, 15]. We do not claim from our study that rivaroxaban is associated with superior mortality, compared with LMWH, but at least there is no evidence of an inferior cancer outcome.

This cohort study also provides some guidance regarding the anticipated confounding situations that frequently arise in the cancer setting, including interruption for procedure, thrombocytopenia, and transient organ dysfunction. Further, our empirical decision to dose-reduce in the elderly was associated with comparable bleeding rates with the overall population and was not associated with a trend towards significant loss of efficacy. Further support for the Clinical Pathway will require expansion of the number of patients treated under these guidelines.

The non-randomized nature of our cohort, which allowed patients and caregivers to choose between rivaroxaban and enoxaparin, could in theory result in selection bias. However this would be unlikely given the small numbers of individuals who were treated with a LMWH at our institution during the period of this cohort study. The primary reason patients were not treated with rivaroxaban was the presence of an active GU or GI tract lesion, which we estimated was <5 % of patients with a new cancer-associated thrombosis, so we do not believe this issue is of material importance for external validity.

This QAI was undertaken to provide guidance on the safety and efficacy of treatment of cancer-associated VTE with rivaroxaban. As we acknowledged, there has been a knowledge gap in this niche, where rivaroxaban has been approved for use, but there have been no prospective randomized studies directly comparing rivaroxaban to LMWH. As such, our objective was to develop and prospectively validate a Clinical Pathway for this situation, providing guidance and reassurance to the providers and patients who have been faced with the burdensome alternative of the cost and inconvenience of LMWH.

Given the small sample size and non-randomized nature of this study, further investigation will be required to establish the safety, efficacy and comparability to a LMWH, the current standard of care. However, there are hurdles before such a clinical trial might be successfully performed. One major issue would be reluctance of patients to participate, given that the oral agent has already been approved for the treatment of VTE in the general population. Until the results of such a direct comparison become available, our data provide reassurance for the use of rivaroxaban in the setting of CAT.

## Electronic supplementary material

Below is the link to the electronic supplementary material.

Supplementary material 1 (DOCX 29 KB)

Supplementary material 2 (DOCX 13 KB)
