# Supplementary material for: Safe and effective use of rivaroxaban for treatment of cancer-associated venous thromboembolic disease: a prospective cohort study
Source: J Thromb Thrombolysis. 2016 Sep 30;43(2):166–71. doi: 10.1007/s11239-016-1429-1 (PMC5318467; doi:10.1007/s11239-016-1429-1)
Supplement: Supplementary file 1 — Supplementary material 1 (DOCX 29 KB) [file 11239_2016_1429_MOESM1_ESM.docx]

**Rivaroxaban Clinical Pathway for Cancer-Associated Venous Thromboembolic Disease: Memorial Sloan Kettering Quality Assurance Initiative.**

**1. Inclusion Criteria**

A. 18 years of age or above

B. Deep vein thrombosis (DVT) of lower extremity and/or pulmonary embolism (PE).

C. Active cancer: Detectable tumor burden (by clinical assessment, imaging, biopsy, serum marker or flow cytometry) AND/OR undergoing chemotherapy AND/OR undergoing radiation therapy.

**2. Relative Contraindications**

A. Active bleeding or a high risk of bleeding (Has-Bled Score of ≥4).

B. Untreated central nervous system primary or metastatic lesion.

C. Previous or planned treatment with thrombectomy, or a fibrinolytic agent.

D. Systolic blood pressure greater than 180 mm Hg or diastolic blood pressure greater than 110 mm Hg.

E. Childbearing potential without proper contraceptive measures, pregnancy, or breast-feeding.

F. Creatinine clearance <30 mL/min.

G. Body weight <50 kg or >150 kg.

H. Clinically significant liver disease (one or more of following):

i. Child-Pugh classification of B or C (see Table 1)

ii. Acute hepatitis, chronic active hepatitis or cirrhosis

iii. At least one of ALT, AST, or total bilirubin > 3 times the upper limit of normal.

I. Concomitant use of any antiplatelet agent other than aspirin 81 mg daily.

J. Ongoing macroscopic hematuria or known untreated urinary tract lesion.

K. Ongoing gastrointestinal bleeding or known untreated gastrointestinal lesion.

L. Expected poor gastrointestinal absorption (any prior surgical alteration of the gastrointestinal tract or medical condition known to result in malabsorption) or need for medications to be administered by tube feedings.

M. Unacceptable Drug Interactions at time of initiation of rivaroxaban:

i. Use of any strong cytochrome P-450 3A4 inhibitor or inducer (see Table 2)

ii. Use of ≥3 moderate cytochrome P-450 3A4 inhibitors or inducers (see Table 2)

iii. Use of ≥2 moderate cytochrome P-450 3A4 inhibitor if also on a P-GP inhibitor (see Table)

iv. Any “X” rated interaction as determined by Lexicomp, except dexamethasone if no more than 3 days every 3 weeks (any dose, any schedule)

v. Other potential significant drug interaction.

N. Any patient with an anticipated period of chemotherapy-induced thrombocytopenia of <25,000/ul for 7 days or longer, will not be eligible. This includes all patients with Acute Myeloid Leukemia, Acute Lymphocytic Leukemia, or Post-Stem Cell Transplant.

**3. Screening process for drug interactions**

A. Upon consideration of initiation of rivaroxaban, the patient’s medication list will be reviewed for interactions as listed above in exclusion criteria.

B. If a drug is found to preclude initiation of rivaroxaban during initial screening, efforts will be made to discontinue or substitute the problematic agent if it is in the best interest of the patient to do so, as determined by the treating hematologist and in concert with the other prescribing physician.

C. Patients will be instructed to contact the Anticoagulation Management Team of any changes to drug regimen or medical situation, to prompt reevaluation of possible drug interactions.

D. If a contra-indicated drug is later added, a similar attempt will be made to discontinue or substitute the agent.

**4. Dosage**

A. Initial Dosing: Rivaroxaban 15 mg PO BID for 3 weeks followed by 20 mg PO daily. Rivaroxaban treatment will tentatively continue as long as active malignancy is present (as defined above). Appropriateness of continued anticoagulation will be based on consideration of ongoing assessment of risk:benefit as determined by the treating physician, as well as reaching a primary endpoint.

B. Adjustment for temporary chemotherapy-induced thrombocytopenia: (Dose reduction of anticoagulation for transient chemotherapy-induced thrombocytopenia is a temporary measure that is standard practice in hematology/oncology. This practice is for patient safety.)

i. Platelet count<25,000/ul: hold rivaroxaban.

ii. Platelet count 25-50,000/ul:

-Rivaroxaban 10 mg PO BID during the first 3 weeks of treatment.

-Rivaroxaban 10 mg PO daily after 3^rd^ week.

iii. Platelet count >50,000/ul: do not adjust the dose of rivaroxaban

iv. If the platelet count is < 50,000/ul from chemotherapy-induced thrombocytopenia, and rivaroxaban dose has been adjusted or held, a CBC with platelet count will be repeated in 5-7 days. If the thrombocytopenia (< 50,000/ul) persists for at least 5-7 days, rivaroxaban will be held. If/when the platelet count recovers to greater than 50,000/ul, the patient may be re-evaluated for resumption of rivaroxaban.

C. Adjustment for age: if age is ≥75 years, decrease the dose of rivaroxaban to 15 mg PO daily if patient would otherwise be on 20 mg PO daily, or 10 mg PO BID if would otherwise be on 15 mg PO BID.

D. Other adjustments: Based on clinical impression of bleeding risk, the dose of rivaroxaban can be decreased or otherwise altered in a manner otherwise not described in the current clinical pathway.

**5. Laboratory testing**

A. Done at baseline (1 week or less before starting rivaroxaban), to be repeated at least every 3 months.

B. Consist of: Complete Blood Count, Comprehensive Metabolic Panel, Prothrombin Time, and Activated Partial Thromboplastin Time.

**6. Management of bleeding episodes**

A. Nuisance bleeding

i. Continue anticoagulant and apply local measures until bleeding resolves.

ii. Assess the potential precipitating factor(s) for the bleeding episode.

iii. Continue on rivaroxaban, if it is judged by Anticoagulation Management Team to be in the patient best interest and patient understands switching to enoxaparin is an alternative.

iv. Attempt to correct any remaining risk factor(s) for bleeding.

B. Clinically relevant non-major bleeding

i. If reasonable, continue anticoagulant and apply local measures until bleeding resolves.

ii. Consider discontinuing rivaroxaban if clinically warranted, after first event.

iii. Discontinue rivaroxaban after 2^nd^ event.

iv. Subsequent management of the thrombosis will be at discretion of Anticoagulation Management Team.

C. Major bleeding

i. Discontinue rivaroxaban.

ii. Best supportive care.

iii. Optionally, administer 4-Factor Prothrombin Complex Concentrate (Kcentra ®). Discuss dose with covering hematologist.

iv. Other measures to control bleeding will be by best judgment of the Anticoagulation Management Team.

v. Further management of the thrombosis will be by best judgment of the Anticoagulation Management Team

**7. Definitions of bleeding**

A. Nuisance bleeding: Overt bleeding not meeting a criterion for major bleeding or clinically relevant non-major bleeding. (i.e. easy bruising, self-limited episode of epistaxis.)

B. Clinically relevant non-major bleeding:

Overt bleeding not meeting a criterion for major bleeding but associated with medical intervention, unscheduled contact with a physician, interruption or discontinuation of treatment, or associated with any other discomfort such as pain or impairment of activities of daily life.

C. Major bleeding (ISTH Criteria[^1^](#_ENREF_1)):

i. Fatal bleeding.

ii. Symptomatic bleeding in a critical area or organ, such as intracranial, intraspinal, intraocular, retroperitoneal, intraarticular or pericardial, or intramuscular with compartment syndrome.

iii. Bleeding causing a fall in hemoglobin level of 2.0 g/dL or more, or leading to transfusion of two or more units of whole blood or red cells.

Table 1: Child-Pugh classification of severity of cirrhosis[^2^](#_ENREF_2)

| **Parameter** | **Points assigned** | | |
| --- | --- | --- | --- |
|  | **1** | **2** | **3** |
| Ascites | Absent | Slight | Moderate |
| Bilirubin | <2 mg/dL (<34.2 micromol/liter) | 2 to 3 mg/dL (34.2 to 51.3 micromol/liter) | >3 mg/dL (>51.3 micromol/liter) |
| Albumin | >3.5 g/dL (35 g/liter) | 2.8 to 3.5 g/dL (28 to 35 g/liter) | <2.8 g/dL (<28 g/liter) |
| Prothrombin time | | | |
| Seconds over control | <4 | 4 to 6 | >6 |
| INR | <1.7 | 1.7 to 2.3 | >2.3 |
| Encephalopathy | None | Grade 1 to 2 | Grade 3 to 4 |

Modified Child-Pugh classification of the severity of liver disease according to the degree of ascites, the serum concentrations of bilirubin and albumin, the Prothrombin time, and the degree of encephalopathy. A total Child-Turcotte-Pugh score of 5 to 6 is considered class A (well-compensated disease); 7 to 9 is class B (significant functional compromise); and 10 to 15 is class C (decompensated disease). These classes correlate with one- and two-year patient survival: class A: 100 and 85 percent; class B: 80 and 60 percent; and class C: 45 and 35 percent.

Table 2: Drugs Interacting with Rivaroxaban*

| **Strong CYP3A4 Inhibitors** | **Moderate CYP3A4 Inhibitors** | **P-GP Inhibitors** | **P-GP Inducers** | **Strong CYP3A4 Inducers** | **Moderate CYP3A4 Inducers** |
| --- | --- | --- | --- | --- | --- |
| Atazanavir | Amiodarone | Afatinib | Phenobarbital | Carbamazepine | Bexarotene |
| Boceprevir | Amprenavir | Amiodarone | Tipranovir | Nevirapine | Bosentan |
| Chloramphenicol | Aprepitant | Bosutinib |  | Oxcarbazepine | Efavirenz |
| Clarithromycin | Atazanavir | Cabozantinib |  | Phenobarbital | Etravirine |
| Conivaptan | Ciprofloxacin | Clarithromycin |  | Phenytoin | Griseofulvin |
| Darunavir | Crizotinib | Conivaptan |  | Primidone | Modafinil |
| Fosamprenavir | Cyclosporine | Crizotinib |  | Rifabutin | Nafcillin |
| Indinavir | Darunavir | Cyclosporine |  | Rifampin |  |
| Itraconazole | Diltazem | Darunavir |  | Rifapentine |  |
| Ketoconazole | Dronaderone | Diltiazem |  | St. John's wort |  |
| Lopinavir/Ritonavir | Erythromycin | Dronaderone |  |  |  |
| Nefazodone | Fluconazole | Erythromycin |  |  |  |
| Nelfinavir | Fosamprenavir | Imatinib |  |  |  |
| Posaconazole | Imatinib | Indinavir |  |  |  |
| Ritonavir | Miconazole | Itraconazole |  |  |  |
| Saquinavir | Sertraline | Ketoconazole |  |  |  |
| Telaprevir | Verapamil | Lapatinib |  |  |  |
| Telithromycin |  | Lopinavir/Ritonavir |  |  |  |
| Voriconazole |  | Nelfinavir |  |  |  |
|  |  | Nilotinib |  |  |  |
|  |  | Ponatinib |  |  |  |
|  |  | Quinidine |  |  |  |
|  |  | Regorafenib |  |  |  |
|  |  | Reserpine |  |  |  |
|  |  | Ritonavir |  |  |  |
|  |  | Saquinavir |  |  |  |
|  |  | Sunitinib |  |  |  |
|  |  | Tacrolimus |  |  |  |
|  |  | Tamoxifen |  |  |  |
|  |  | Vandetanib |  |  |  |
|  |  | Verapamil |  |  |  |

*Drugs in red are listed in 2 columns at the same time, i.e. they are both moderate CYP3A4 inhibitors and P-GP inhibitors. Collated by MSK pharmacy department after a review of available literature.

**References**

1. Schulman S, Kearon C, Subcommittee on Control of Anticoagulation of the S, et al: Definition of major bleeding in clinical investigations of antihemostatic medicinal products in non-surgical patients. J Thromb Haemost 3:692-4, 2005

2. Pugh RN, Murray-Lyon IM, Dawson JL, et al: Transection of the oesophagus for bleeding oesophageal varices. Br J Surg 60:646-9, 1973
