# Supplementary material for: Safe and effective use of rivaroxaban for treatment of cancer-associated venous thromboembolic disease: a prospective cohort study
Source: J Thromb Thrombolysis. 2016 Sep 30;43(2):166–71. doi: 10.1007/s11239-016-1429-1 (PMC5318467; doi:10.1007/s11239-016-1429-1)
Supplement: Supplementary file 2 — Supplementary material 2 (DOCX 13 KB) [file 11239_2016_1429_MOESM2_ESM.docx]

**Appendix II: Key Words**

Thrombosis:

pe

<LineFeed>pe

pe<LineFeed>

<LineFeed>pe<LineFeed>

pes

<LineFeed>pes

pes<LineFeed>

<LineFeed>pes<LineFeed>

clot

dvt

filing defect

pulm embol

pulmonary embol

thrombi

thrombo

thrombus

Bleeding:

anticoagulation complication

bleed

blood in the stools

blood in the urine

blood loss

blood per rectum

blood tinged

bloody

coffee ground emesis

epistaxis

haemorrhag

hematemesis

hematochezia

hematoma

hematuria

hemopericardium

hemoperitoneum

hemoptysis

hemorrhag

hemothorax

melena

rectorrhagia

tarry stools
